# Supplementary material for: Brief Investigation: Investigating the role of phosphodiesterase Pde2 in coordinating the yeast Environmental Stress Response
Source: bioRxiv. 2026 May 22:2026.05.20.726645. Preprint. [Version 1] doi: 10.64898/2026.05.20.726645 (PMC13228386; doi:10.64898/2026.05.20.726645)
Supplement: Supplement 1 [file NIHPP2026.05.20.726645v1-supplement-1.pdf]

bioRxiv preprint doi: https://doi.org/10.64898/2026.05.20.726645; this version posted May 22, 2026. The copyright holder for this preprint (which was not certified by peer review) is the author/funder, who has granted bioRxiv a license to display the preprint in perpetuity. It is made available under a CC-BY 4.0 International license.

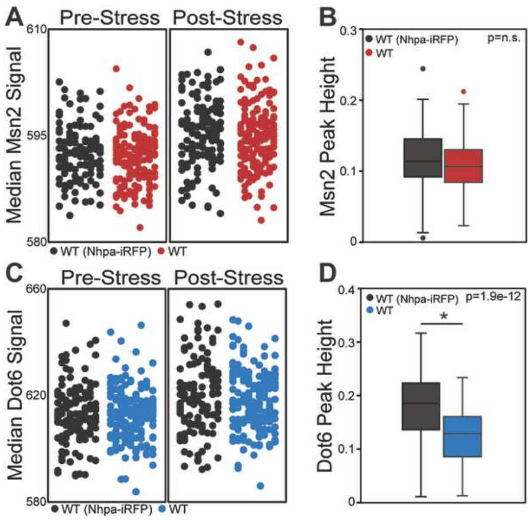

Figure S1. iRFP expression can affect peak height measurement in the GFP channel. Control experiments mixed two wild-type strains, both expressing Msn2-mCherry and Dot6-GFP but where only one strain carried the additional iRFP marker. **A and C**) Median pixel intensity of Msn2-mCherry (A) or Dot6-GFP (C) and **B and D**) nuclear translocation peak heights of Msn2-mCherry (B) and Dot6-GFP (D) are shown for the two strains. Cells carrying the iRFP marker reproducibly showed a larger apparent nuclear translocation signal in the GFP channel (see **D**), indicating an artifact of the iRFP marker. P-values based on Wilcoxon rank-sum test.
